# Genetic diversity and population structure of malaria vector mosquitoes *Anopheles subpictus, Anopheles peditaeniatus,* and *Anopheles vagus* in five districts of Sri Lanka

**DOI:** 10.1186/s12936-018-2419-x

**Published:** 2018-07-20

**Authors:** Thilini C. Weeraratne, Sinnathambi N. Surendran, Catherine Walton, S. H. P. Parakrama Karunaratne

**Affiliations:** 1 Department of Zoology, Faculty of Science, University of Peradeniya, Peradeniya, Sri Lanka; 2 Department of Zoology, Faculty of Science, University of Jaffna, Jaffna, Sri Lanka; 3 School of Earth and Environment, Faculty of Science and Engineering, University of Manchester, Manchester, UK

**Keywords:** *Anopheles subpictus*, *Anopheles peditaeniatus*, *Anopheles vagus*, Population genetic structure, *COI*, Sri Lanka

## Abstract

**Background:**

Although Sri Lanka is considered as a malaria-free nation, the threat of re-emergence of outbreaks still remains due to the high prevalence and abundance of malaria vectors. Analysis of population genetic structure of malaria vectors is considered to be one of the vital components in implementing successful vector control programmes. The present study was conducted to determine the population genetic structure of three abundant malaria vectors; *Anopheles subpictus* sensu lato (s.l.)*, Anopheles peditaneatus* and *Anopheles vagus* from five administrative districts in two climatic zones; intermediate zone (Badulla and Kurunegala districts) and dry zone (Ampara, Batticoloa and Jaffna districts) of Sri Lanka using the mitochondrial gene, *cytochrome c oxidase subunit I* (*COI*).

**Methods:**

Adult mosquitoes of *An. subpictus* s.l.*, An. peditaeniatus,* and *An. vagus* were collected from five study sites located in five districts using cattle baited traps and backpack aspirators. Representative samples of each species that were morphologically confirmed were selected from each locality in generating *COI* sequences (> 6 good quality sequences per species per locality).

**Results:**

*Anopheles subpictus* s.l. specimens collected during the study belonged to two sibling species; *An. subpictus* ‘A’ (from all study sites except from Jaffna) and *An. subpictus* ‘B’ (only from Jaffna). The results of haplotype and nucleotide diversity indices showed that all the three species are having high genetic diversity. Although a high significant pairwise difference was observed between *An. subpictus ‘*A’ and ‘B’ (*F*_*st*_> 0.950, *p *< 0.05), there were no significant genetic population structures within *An. peditaeniatus*, *An. vagus* and *An. subpictus* species A (*p *> 0.05), indicating possible gene flow between these populations.

**Conclusions:**

Gene flow among the populations of *An. peditaeniatus*, *An. vagus* and *An. subpictus* species A was evident. Application of vector control measures against all mosquito species must be done with close monitoring since gene flow can assist the spread of insecticide resistance genes over a vast geographical area.

**Electronic supplementary material:**

The online version of this article (10.1186/s12936-018-2419-x) contains supplementary material, which is available to authorized users.

## Background

Knowledge on the population genetic structure of mosquito vectors of disease is vital in understanding their vectorial capacity, in increasing the efficiency of existing vector control programmes and in implementing novel vector control strategies [1–5]. For these reasons, population genetic structures of *Anopheles* mosquitoes, many species of which are vectors of malaria, have been extensively studied, e.g. *Anopheles arabiensis* [6, 7], *Anopheles baimaii* [8], *Anopheles culicifacies* [9], *Anopheles dirus* [3, 10, 11], *Anopheles funestus* [12], *Anopheles gambiae* [13, 14], *Anopheles maculatus* [15], *Anopheles minimus* [16], *Anopheles sinensis* [17–19] and *Anopheles stephensi* [20, 21].

Studies have shown geographical barriers to be a major determinant of genetic structure of mosquitoes compared to the geographic distance [4, 12, 13, 15, 18]. However, geographic distance and barriers to gene flow can operate in combination to generate population genetic structure e.g. *An. sinensis* populations in China [17]. Moreover, in Thailand, the genetic structure of *Aedes aegypti* populations has been shown to be influenced by intense vector control activities [5].

Several mitochondrial DNA (mtDNA) regions have been used as successful genetic markers in barcoding of mosquitoes and, in analyzing the genetic diversity and genetic structure of populations. Among these markers, *cytochrome c oxidase subunit I* (*COI*) has been the most extensively used marker in studies on the genetic structure of mosquitoes, including *An. sinensis* [17], *An. baimaii* [8], *An. dirus* [10, 11, 22], *An. lesteri* [23], *An. darling* [24], *An. stephensi* [21] and *Aedes albopictus* [25].

The mosquito fauna of Sri Lanka is represented by 141 species, of which 23 belong to the genus *Anopheles*. Species *An. culicifacies* and *An. subpictus* are considered respectively as primary and secondary vectors of malaria [26–28]. Whereas *Anopheles aconitus*, *Anopheles annularis*, *Anopheles barbirostris*, *Anopheles nigerrimus*, *Anopheles pallidus*, *Anopheles peditaneatus*, *Anopheles tessellatus*, *Anopheles vagus* and *Anopheles varuna* are considered as potential malaria vectors in Sri Lanka [29, 30]. Also *An. stephensi*, one of the major malaria vectors in India was recently discovered from northwestern coasts of Mannar in Sri Lanka [31, 32]. Although the World Health Organization declared Sri Lanka a malaria-free nation in 2016, there is a high risk of reemergence of the disease with an introduction of the parasite, especially through travelers from malaria endemic countries, as the vectors are available throughout the country [33]. Currently the country keeps the vectors suppressed mainly through the use of a combination of organophosphates and pyrethroids in vector control programmes.

Continuous exposure to insecticides over a long period of timed imposes a great selection pressure to develop insecticide resistance in mosquito populations. Both the major vectors *An. culicifacies* and *An. subpictus*, and several other potential vector species including *An. peditaeniatus* and *An. nigerrimus* have developed resistance to a range of insecticides from all the major groups; organochlorines, organophosphates, carbamates and pyrethroids [34]. It has been shown that the gene flow play an important role in the spread of resistance genes in mosquito populations [35–37]. Therefore, resistance genes developed in a vector population of one particular area can be spread effectively into other areas of the country through the gene flow.

Among the malaria vectors found in Sri Lanka, *An. culicifacies*, *An. subpictus*, *An. annularis* and *An. barbirostris* occur as species complexes [33]. *Anopheles subpictus* exists as a sibling species complex and studies have shown the occurrence of two genetically distinct entities of this species; *An. subpictus* ‘A’ and *An. subpictus* ‘B’ [26, 33]. Of the two members of Culicifacies complex present in Sri Lanka, *An. culicifacies* species E is the vector of malaria parasite whereas B is a non-vector. Species E always has shown relatively high resistance to commonly used insecticides than species B [9]. Population genetic structure analysis of *An. culicifacies* E using microsatellite data has shown the effect of geographic barriers on the genetic variation of this species [38]. As sibling species can have different feeding habits, behavior patterns, disease transmission rates, similar control measures might not be effective against different sibling species.

Hence, studies on genetic diversity and population structure of malaria vectors is important in implementing successful vector control programmes against the reemergence of malaria in the country. Few studies have been carried out to determine the population genetic structure of Sri Lankan *An. culicifacies* previously [9, 38]. This study aims to analyse the population genetic structure of another three important malaria vectors *An. subpictus*, *An. peditaneatus*, and *An. vagus* using the mitochondrial gene, *cytochrome c oxidase subunit I* (*COI*), for the first time in Sri Lanka.

## Methods

### Study sites and mosquito collection

**Fig. 1:**
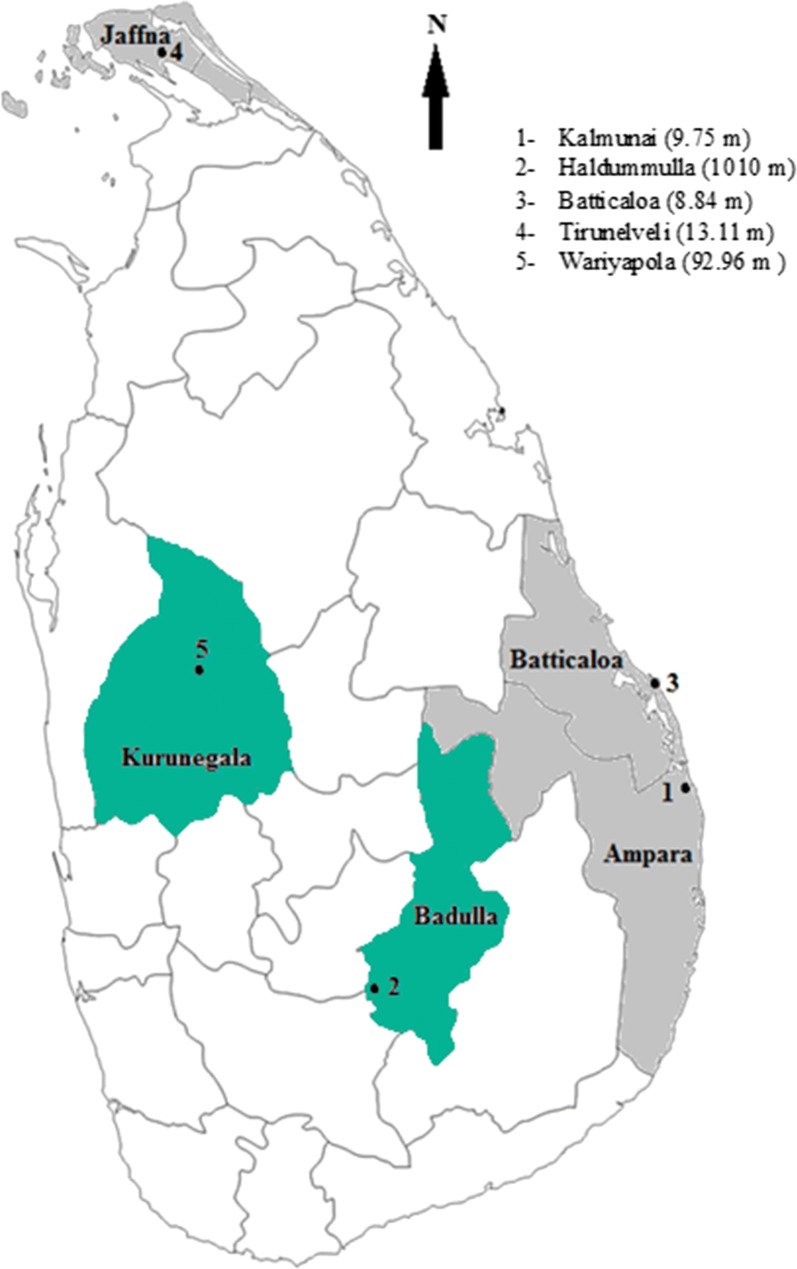
Five study sites located in each district where *An. peditaeniatus*, *An. subpictus* s.l. and *An. vagus* mosquitoes were collected for the population genetic structure analysis (elevations of the study sites are given in parentheses; green—intermediate zone, grey—dry zone)

Mosquitoes were collected from five districts of Sri Lanka. A single locality was selected from each district; Kalmunai in Ampara district, Haldummulla in Badulla district, Batticaloa in Batticaloa district, Tirunelveli in Jaffna district, Wariyapola in Kurunegala district (Fig. 1). These sites are located in the following climatic zones in Sri Lanka i.e. Haldummulla in up country intermediate zone (> 900 m elevation 1750–2500 mm rainfall); Wariyapola in low country intermediate zone (0–300 m elevation, 1750–2500 mm rainfall); Kalmunai, Batticaloa and Tirunelveli in low country dry zone (0–300 m elevation, < 1750 mm rainfall with a distinct dry period) (Fig. 1). The highest geographic distance was between Tirunelveli and Kalmunai (322 km) and lowest was between Batticaloa and Kalmunai (37 km) study sites.

Adult mosquitoes were collected monthly using cattle baited traps (one trap in each study site) and backpack aspirators from January 2014 to July 2015. Samples were collected from 2 to 3 points from each study site. These localities were selected based on previous study results where high abundance of these vectors was reported in all five selected study sites [33]. Dried specimens were morphologically identified into species level using standard taxonomic keys [39] and stored for molecular characterization. A representative randomly selected samples of each species from each locality was selected for sequencing.

### DNA extraction, polymerase chain reaction (PCR) and sequencing

Genomic DNA was extracted from head and thoracic regions of each morphologically identified individuals using nexttec™ DNA Isolation Kits (Nexttec Biotechnologies GmbH, Leverkusen, Germany), according to the manufacturer’s protocol.

A region of the *COI* gene was amplified using forward primer C1-J-1718 (5′-GGAG GATTTGGAAATTGATTAGTTCC-3′) and reverse primer C1-N-2191 (5′ CCCGGTAAAATTAAAATATAAACTTC-3′) [40]. Each amplification was performed in 15 µl that included 1 µl of DNA template, 1.5 µl of 10× KAPA buffer A, 0.12 µl of KAPA taq, 0.12 µl of 2.5 mM dNTP mix, 0.75 µl of 50 mM MgCl_2_, 0.51 µl of each primer (10 mmol) and 10.49 µl of ddH_2_O. The PCR parameters were 95 °C for 5 min and 35 cycles of 94 °C for 30 s, 51 °C and 72 °C for 45 s, followed by a final extension step of 72 °C for 10 min. PCR products were run in 1.5% agarose gel stained with Medori green and visualized in a gel imaging system.

PCR products showing positive clear bands were purified using QIAquick^®^ PCR Purification kits according to the manufacturers’ protocol. A minimum of six PCR positive samples of each species from each district were sequenced bidirectionally at Source Bioscience, Nottingham, United Kingdom.

### Statistical analysis

The trace files/chromatograms of *COI* sequences (a minimum of 6 sequences for each species from each district) were manually edited using BioEdit software. Sequences of low quality were excluded and a minimum of 6 good quality sequences from each species from each locality were used for data analysis. After trimming the *COI* sequences to remove ambiguous sites, final fragments of 403 bp in *An. peditaeniatus* and *An. subpictus* and, 423 bp in *An. vagus* were used in the genetic diversity and population genetic structure analysis. Once the alignment was completed, sequences were compared with the publicly available sequence data in GenBank using BLAST [41] and the BOLD interface [42] to confirm species identification. Amino acid sequences were inferred to check for the presence of stop codons. Number of haplotypes (h), genetic diversity indices [Haplotype Diversity Index (Hd) and Nucleotide Diversity Index (Pi)] and, Neutrality tests (Tajima’s D and Fu’s Fs) were performed in DNA Sequences Polymorphism software (dnaSP) (version 5.1.10). Pairwise differences and population structures of each species were evaluated by analysis of molecular variance (AMOVA) in Arlequin 3.11 and significance was evaluated based on 10,000 permutations. Based on the number of nucleotide differences, haplotype networks of these three species were constructed using Network software 5.0.0.1 to determine the interrelationship between haplotypes.

## Results

Translated amino acid sequences revealed that there are no frame shifts or stop codons in all the edited sequences, indicating the mitochondrial origin of the DNA. Comparison of *COI* sequences of *An. peditaeniatus* and *An. vagus* with the publicly available sequences completely agreed with our morphological identification. The morphologically identified *An. subpictus* s.l., specimens belonged to two genetic entities. All the specimens from Jaffna belonged to *An. subpictus* species B while specimens from the other four sites belonged to *An. subpictus* species A. The haplotype diversities (Hd) and nucleotide diversities (Pi) were similarly high for all species except for *An. subpictus* species B which reported relatively low Hd (0.666) and Pi (0.002) (Table 1). According to neutrality test results, both Tajima’s D and Fu’s Fs values were not significant in any of the species (*p *> 0.100) (Table 1).

**Table 1:** Genetic diversity indices, neutrality test values and GenBank accession numbers for *An. subpictus* s.l., *An. peditaeniatus* and *An. vagus*

Species	S	h	Hd (± SD)	Pi (± SD)	D	Fs	GenBank accession nos.
*An. subpictus* species A	11	14	0.880 ± 0.002	0.006 ± 0.001	− 0.626	− 6.109	KX644166–KX644181
*An. subpictus* species B	1	2	0.666 ± 0.031	0.002 ± 0.000	1.224	0.625	KX644182–KX644183
*An. peditaeniatus*	14	10	0.848 ± 0.045	0.006 ± 0.001	− 0.990	− 2.209	KX644156–KX644165
*An. vagus*	25	12	0.849 ± 0.051	0.008 ± 0.002	− 1.522	− 2.267	KX668152–KX668163

*S* no. of polymorphic sites, *h* number of haplotypes, *Hd* haplotype diversity, *Pi* nucleotide diversity, *D* Tajima’s D, *Fs* Fu’s Fs

**Fig. 2:**
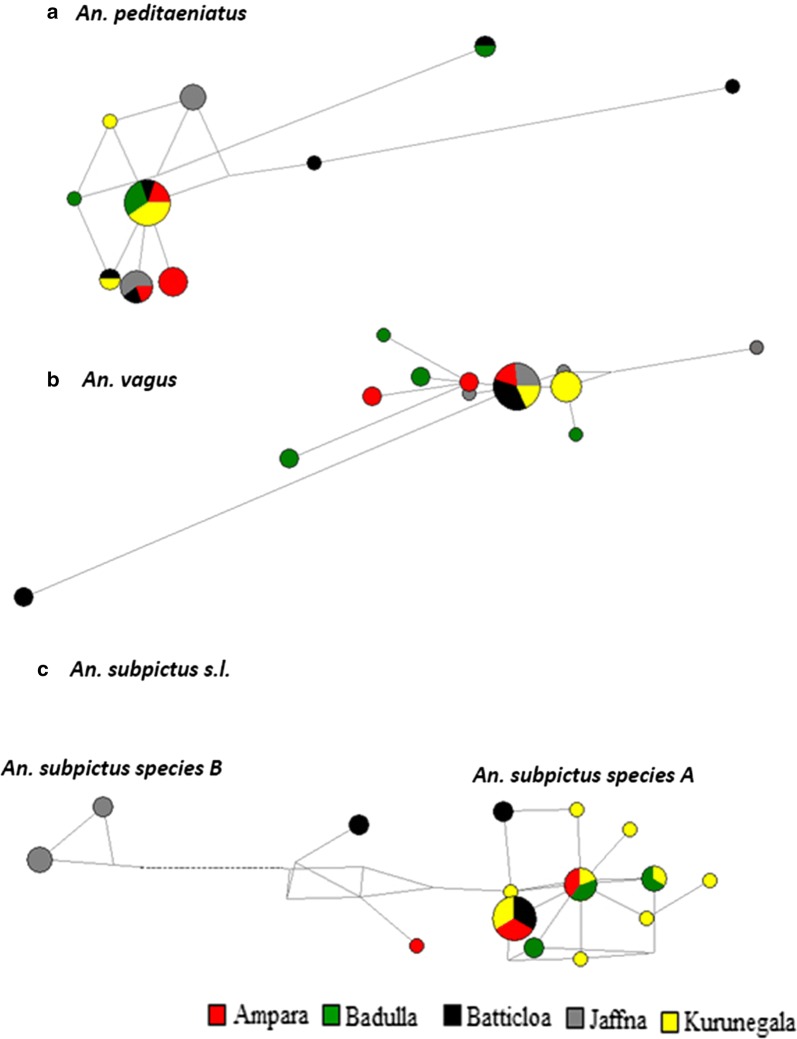
Haplotype networks generated using Network 5.0.0.1 for **a** *An. peditaeniatus*, **b** *An. vagus* and **c** *An. subpictus* s.l. collected from five geographical locations in Sri Lanka. Each haplotype is represented by a circle and the size of the circle is proportional to the number of individuals with each haplotype. Geographical localities are colour coded

*Anopheles peditaeniatus* showed the highest number of haplotype sharing among the five studied localities (4 shared haplotypes) followed by *An. subpictus* species A (3 shared haplotypes) and *An. vagus* (1 shared haplotype). *Anopheles subpictus* species B was present only in the Jaffna study site. The most dominant haplotype of *An. peditaeniatus* (33.33% of the total number of haplotypes) and *An. vagus* (35.48% of the total number of haplotypes) were shared among 4 localities while that of *An. subpictus* species A (25.71% of the total number of haplotypes) was shared only between three localities as shown in Fig. 2.

Unlike the haplotype network drawn for *An. peditaeniatus* and *An. vagus* the haplotype network of *An. subpictus* s.l. formed two distinct clusters; one representing 16 haplotypes of *An. subpictus* species A and the other representing 2 haplotypes of *An. subpictus* species B (Fig. 2c).

The pairwise comparison of population differentiation is presented in Table 2. *Anopheles subpictus* species B population from Jaffna showed a very high significant pairwise difference with four *An. subpictus* species A populations with *F*_*ST*_ values always greater than 0.950 (*p *< 0.05) (Table 2). Population pairwise *F*_*ST*_ values within *An. peditaeniatus, An. subpictus* species A and *An. vagus* were not significant indicating an absence of genetic differentiation among populations within these species (Table 2).

**Table 2:** Pairwise *F*_*ST*_ values obtained for *An. subpictus* s.l., *An. peditaeniatus* and *An. vagus* of the study

District	Jaffna	Kurunegala	Badulla	Ampara	Batticaloa
*An. subpictus* s.l.					
Jaffna	0.000				
Kurunegala	0.971*	0.000			
Badulla	0.975*	0.127	0.000		
Ampara	0.971*	− 0.011	0.160	0.000	
Batticaloa	0.957*	0.044	0.232	− 0.063	0.000
*An. peditaeniatus*					
Jaffna	0.000				
Kurunegala	0.026	0.000			
Badulla	0.113	0.190	0.000		
Ampara	0.046	0.040	0.042	0.000	
Batticaloa	0.000	0.074	0.011	0.118	0.000
*An. vagus*					
Jaffna	0.000				
Kurunegala	0.006	0.000			
Badulla	0.114	0.128	0.000		
Ampara	0.167	0.191	0.001	0.000	
Batticaloa	0.121	0.150	0.137	0.103	0.000

* Significant (*p *< 0.05)

AMOVA was conducted to estimate the genetic structure variation among populations of each species and the results obtained are shown in Additional file 1. According to the variations estimated for *An. peditaeniatus*, *An. vagus*, and *An. subpictus* species A, a significant variation was observed among individuals within populations (percentage variation; 101.99% for *An. peditaeniatus* and 102.29% for *An. vagus* and 100.47% for *An. subpictus* species A) (*p *< 0.05).

## Discussion

Suppression of the malaria vector population is the most effective way of preventing the re-emergence of malaria outbreaks in Sri Lanka. The present study was conducted to analyze population genetic structure of three abundant malaria vectors, as this knowledge is important in planning future vector control programmes of Sri Lanka.

The present study also reports the presence of two genetic entities of *An. subpictus*; “species A” and “species B” confirming the results of the previous study on barcoding of Anopheline mosquitoes from the same study sites [33]. High *F*_*st*_ values obtained during the comparison between *An. subpictus* species A and species B populations, indicated that these two are genetically distinct from each other.

Both pairwise comparisons (*F*_*st*_ values) and the analysis of molecular variance (AMOVA) showed that there is no genetic structure variation in *An. peditaeniatus*, *An. vagus* and *An. subpictus* species A populations used during the current study. These species showed haplotype sharing between the five populations and it was highest for *An. peditaeniatus*. A mechanism of mixing of these mosquito populations from different geographical areas and possible gene flow is evident by these observations even though a considerably high geographic distance, ranging from 32 to 322 km, is present between these study sites. Sri Lanka is an island with 103 rivers basins and all these three species of mosquitoes breed in variety of freshwater habitats, which are connected to these riverine systems one way or the other. Further, all these rivers start from the mountainous areas at the center of the country and flow through the three climatic zones (wet, intermediate and dry zone) before joining the sea. Hence, the sites of the current study are connected by mosquito habitats, which allow gene flow between these localities. Further, the rainfall experienced by these study sites also build up this connection between the breeding sites.

Sri Lanka is an island with a relatively small land area and there were no major geographical barriers between the studied localities. Hence, regardless of the geographic distance the possibility of gene flow between the study sites is considerably higher. *Anopheles maculatus* populations that have been separated by 50 km have shown limited gene flow in the presence of geographic barriers while in the absence of geographic barriers free gene flow has been observed even between populations 650 km apart [15]. Several other studies related to mosquitoes have also reported absence of correlation between genetic isolation and geographic distance [4, 5, 7, 18, 19].

However, a study using *COI* marker and microsatellites has shown that the geographic distance has an effect on the genetic structure variation of Sri Lankan *An. culicifacies* populations [9]. Although the central mountain range of Sri Lanka has acted as a barrier for the gene flow of *An. culicifacies* E, it was not a barrier for *An. peditaeniatus*, *An. vagus* and *An. subpictus* species A [38]. Therefore, it appears that the relationship between geographic distance and the population genetic structure of anophelines depends on the type of the species probably due to species wise variations in breeding habitats, breeding patterns and behaviour.

Continuity of mosquito breeding sites supported by the absence of geographical barriers can be considered as the main reason for the maintenance of gene flow between the *An. peditaeniatus*, *An. vagus* and *An. subpictus* species A populations in Sri Lanka. Regular monitoring of population genetic structure of malaria vectors is important in developing effective vector control strategies to address the possible impact made by the spread of vital genes such as insecticide resistance genes through vector populations.

## Conclusions

*Anopheles subpictus* s.l. collected from five Sri Lankan districts belonged to two genetically distinct species *An. subpictus* species A and *An. subpictus* species B. Gene flow was evident even between geographically distant populations of *An. peditaeniatus*, *An. vagus* and *An. subpictus* species A perhaps due to absence of geographic barriers and the continuity of habitats. Results also validated the use of *COI* gene as a tool in understanding gene flow of anophelines in Sri Lanka.

## Additional file

**Additional file 1.** Results of *COI* genetic structure variations estimated using AMOVA for *An. subpictus*, *An. peditaeniatus* and *An. vagus* collected from five geographical locations in Sri Lanka.

## Abbreviations

*COI*: *Cytochrome c oxidase subunit I*
mtDNA: mitochondrial DNA
Hd: Haplotype Diversity Index
Pi: Nucleotide Diversity Index
dnaSP: DNA Sequences Polymorphism software
AMOVA: analysis of molecular variance
