# Supplementary material for: Genetic diversity and population structure of malaria vector mosquitoes Anopheles subpictus, Anopheles peditaeniatus, and Anopheles vagus in five districts of Sri Lanka
Source: Malar J. 2018 Jul 20;17:271. doi: 10.1186/s12936-018-2419-x (PMC6053832; doi:10.1186/s12936-018-2419-x)
Supplement: Supplementary file 1 — Additional file 1. Results of COI genetic structure variations estimated using AMOVA for An. subpictus, An. peditaeniatus and An. vagus collected from five geographical locations in Sri Lanka. [file 12936_2018_2419_MOESM1_ESM.docx]

**Additional file 1.**

Additional file 1. Results of *COI* genetic structure variations estimated using AMOVA for *An. subpictus*, *An. peditaeniatus* and *An. vagus* collected from five geographical locations in Sri Lanka.

| Source of variation | df | Sum of squares | Variance components | Percentage of variation | *p* |
| --- | --- | --- | --- | --- | --- |
| ***An. subpictus species A*** | 3 | 5.694 | -0.07446 | -0.47 | 0.717 |
| Among populations | 13 | 23.565 | 1.22494 | 100.47 | 0.000* |
| Among individuals within populations |  |  |  |  |  |
|  |  |  |  |  |  |
| ***An. peditaeniatus*** |  |  |  |  |  |
| Among populations | 4 | 10.333 | -0.21714 | -1.99 | 0.789 |
| Among individuals within populations | 12 | 27.833 | 1.57547 | 101.99 | 0.000* |
|  |  |  |  |  |  |
| ***An. vagus*** |  |  |  |  |  |
| Among populations | 4 | 17.055 | -0.17333 | -2.29 | 0.494 |
| Among individuals within populations | 10 | 36.429 | 2.04000 | 102.29 | 0.000* |

df- Degree of freedom, * Significant (*p*<0.05).
